# Basal Cell Carcinoma Perineural Invasion and Suggestive Signs of Perineural Invasion—Findings and Perspectives

**DOI:** 10.3390/life13061406

**Published:** 2023-06-17

**Authors:** Elena Niculet, Carmen Bobeica, Cristian Onisor, Gabriela Gurau, Aurel Nechita, Diana Sabina Radaschin, Dana Tutunaru, Laura Bujoreanu-Bezman, Alin Laurentiu Tatu

**Affiliations:** 1 Department of Morphological and Functional Sciences, Faculty of Medicine and Pharmacy, “Dunarea de Jos” University of Medicine and Pharmacy, 800008 Galati, Romania; 2 Multidisciplinary Integrated Center of Dermatological Interface Research MIC-DIR (Centrul Integrat Multidisciplinar de Cercetare de Interfata Dermatologica—CIM-CID), “Dunărea de Jos” University, 800201 Galati, Romania; 3 Department of Pediatrics, ‘Sf. Ioan’ Clinical Hospital for Children, 800487 Galati, Romania; 4 Clinical Medical Department, Faculty of Medicine and Pharmacy, “Dunarea de Jos” University of Medicine and Pharmacy, 800008 Galati, Romania; 5 Dermatology Department, “Sfanta Cuvioasa Parascheva” Hospital of Infectious Diseases, 800179 Galati, Romania; 6 Department of Pharmaceutical Sciences, “Dunarea de Jos” University of Medicine and Pharmacy, 800008 Galati, Romania

**Keywords:** basal cell carcinoma, perineural invasion, perineural inflammation, perineural chronic inflammation

## Abstract

Basal cell carcinoma (BCC) is a malignant tumor with a rising incidence and is the beneficiary of several innovative evaluation techniques. Histopathology remains the gold standard for assessment, having the possibility of addressing multiple high-risk factors such as perineural invasion (PNI). The current study included a number of 244 BCC patients and targeted the identification of positive PNI and its suggestive signs, and whether they correlated or not with other high-risk tumor signs. PNI was found in 20.1% of patients, with 30.7% of patients having perineural chronic inflammation (PCI), which is a suggestive sign of PNI. PNI was also found in larger tumors, with deeper Clark levels, in high-risk BCCs and high-grade tumors. PNI and PCI are both important for pathology reporting, aiding in treatment choice and further patient management, with possibly positive outcomes concerning morbidity and mortality.

## 1. Introduction

Basal cell carcinoma (BCC), a malignant tumor with a keratinocyte origin, is a non-melanoma skin cancer (NMSC) with increasing incidence worldwide and has the capacity to develop from a benign lesion (verruca vulgaris) or an inflammatory dermatopathology (such as systemic sclerosis) by develops from the basal cells of the hair follicle or from those found at the level of the inter-follicular epidermis [1,2,3,4]. In the United States of America (USA), BCC has reached top incidences (an increasing trend paralleling that of skin infections) of as high as 50% of all types of cancers, but with low mortality and high morbidity rates (if it is left untreated for a long period of time) [5,6]; it is one of the most frequent malignant tumors found among the white population [7], with a lifetime risk of developing such a tumor of approximately 28% for women and as high as 39% for men [8].

The clinical aspects of BCCs are highly varied, with increased difficulty in making a positive diagnosis due to its myriad facets. When dealing with a BCC clinical lesion, the differential diagnosis sets a wide net of possibilities, spanning from inflammatory pathologies to benign tumors, and even to malignant ones. BCC can be clinically differentiated from either inflammatory (psoriasis or other types of dermatitis) or benign lesions (such as fibroepithelial polyp, seborrheic keratosis, or follicular processes), or from Spitz-Reed nevi, to malignant ones such as melanoma or SCC [1,5,9]. The accuracy of a BCC’s clinical diagnosis has been drastically improved with the help of newer investigative tools such as dermoscopy, reflectance confocal microscopy, optical coherence tomography [5,10], Raman spectroscopy, high-resolution ultrasonography and terahertz pulse imaging, which have been found to yield better results with regard to the tumor’s margins and its depth of invasion, as important clues for its future successful surgical excision [1].

Histopathology evaluation has long been considered as being the gold-standard for a BCC’s positive diagnosis, while Mohs micrographic surgery (MMS) is the gold-standard with regard to the therapeutic approach (especially in tumors arising in the head and neck region, and more specifically the face, which also benefits from this procedure’s cosmetic approach, without wide excision and extensive local impact) [11,12]. A surgical approach, be it classical excisional or MMS, offers, on the one hand, an important view on the tumor via the possibility of evaluating residual nests, and on the other hand a low rate of tumor recurrence after excision. This being said, treatment is patient-oriented and established according to the individual’s characteristics (general health condition, location of tumor, recurrence risk) [13]. Tumors that are diagnosed at an early stage or superficial basal cell carcinomas may benefit from local treatments such as laser, cryotherapy, photodynamic therapy, retinoids or topical agents (5-Fluorouracil or imiquimod cream) [8]; cases of superficial BCCs (a low-risk tumor) include the single BCC subtype approved for treatment with 5-fluorouracil, imiquimod and ingenol mebutate [14,15,16,17]. There are some general indications regarding BCC topical treatment, being indicated for superficial tumors, small and/or multiple lesions, the elderly, immune suppressed patients, those with surgery phobia and those with cosmetic sequelae [17]. Large, inoperable tumors might benefit from radiotherapy as an alternative treatment. Alternatively, tumors that are not suitable for radiotherapy might receive vismodegib, an inhibitor of an intracellular signaling pathway which has been approved since 2014 [14].

PNI was simply defined in 1985 by Batsakis as tumor cells being present inside, around and through a nerve [18]. In the past, the theory of nerve sheath angiolymphatic invasion was taken into consideration as an explanation for such phenomena, but it was later disregarded due to the fact that in such a location there is no lymphatic circulation [19]. PNI represents tumor extension along the nerve fiber through the path of least resistance of the tissue planes, by direct growth. Once the space found in-between the nerve sheath and the nerve fiber itself is reached by the neoplastic malignant process, it can extend freely, without finding any resistance, from the smallest nerve fibers to the central subarachnoid space. The nerve fibers themselves are not affected by the growth that takes place, due to the increased elasticity of the perineural and endoneural spaces (this characteristic also explains the lack of symptoms which accompany PNI, until the late stages); nerve degeneration due to the pressure exerted by the malignancy takes place only in confined areas, which restricts growth. This elasticity is the reason why medicine has its malevolent “surprises”, including such reports sometimes occurring as the one of a tumor extending 14 cm without it being symptomatic [20].

Perineural invasion (PNI) is a tumor feature which indicates a poor patient prognosis, being a mechanism for tumor dissemination; it is a feature of high-risk tumors (due to the decreased chances of tumor eradication that PNI implies) which needs to be detected as early as possible in order to avoid the onset of symptoms and the possible involvement of multiple nerve fascicles with deeper tumor extension [21,22,23]. As well as SCC, another keratinocytic malignancy, BCC, can be considered a neurotropic type of cancer, but with much less frequency [23]. There is a difference between clinical PNI, which implies the radiological involvement and/or clinical symptoms of PNI, and incidental PNI, found in histopathology reporting (which might precede the onset of clinical involvement, and reporting it could lead to early treatment) [24]. Chronic perineural inflammation is considered to be a positive sign for PNI [21,25,26]; it indicates that the particular nerve branch is pathologically involved. Our study on 244 BCC patients tried to identify the positive PNI and the suggestive signs thereof, and whether they might be correlated or not with other high-risk tumor signs.

## 2. Materials and Methods

Design: The current study is a retrospective one which was carried out in the Pathology Laboratory of “Sfântul Apostol Andrei” Emergency Clinical Hospital of Galati, Romania, and included 244 patients with a positive histopathological diagnosis of BCC over the span of a two-year period, from 2019 (165 patients) to 2020 (79 patients). We used the hospital’s electronic database, the pathology laboratory’s registers and the histopathology slides in order to select the patients eligible for study inclusion.

Inclusion criteria: All patients with skin tumors diagnosed from 2019 to 2020 with a positive histopathological BCC diagnosis were included, after revising the slides and confirming the diagnosis.

Exclusion criteria: This study deals with BCC cases, and all patients without this pathology were excluded from this study in the two years taken into consideration. At the same time, histopathologically confirmed cases of BCC which, during revision, did not meet the criteria for a positive diagnosis, were also excluded.

Main variables collected: Perineural invasion was considered according to the definition of Liebig et al. 2009 (also valid in 2018, Schmidt et al.): “tumor in close proximity to nerve and involving at least 33% of its circumference or tumor cells within any of the 3 layers of the nerve sheath” [23,27]. Maximum tumor dimension: the tumor’s largest dimension on the slide was measured (in micrometers), with the help of a microscope fitted with camera and software. The tumor’s Clark levels were considered to be those that are already in use for melanoma cases: level I—confined to epidermis, II—superficial dermis invasion, III—superficial-deep dermis interface invasion, IV—deep dermis invasion, V—hypodermis invasion [28].

Ethics: All patients included in the study have given their informed consent, which was registered in their medical charts. The study was conducted in accordance with the Declaration of Helsinki, and approved by the Ethics Committee of the “Sfantul Apostol Andrei” Emergency Clinical Hospital of Galati, Romania, with the approval number 2810 from 2 February 2023.

Statistics: The selected patient data were introduced in an Excel table, then imported and processed with the help of SPSS 27.0. The data were sorted into categories and then the frequency distribution was carried out. The possible influences between the data were investigated with the help of the Fisher and Chi-squared tests. Descriptive statistics were used for the quantitative variables and the possible differences were investigated using parametric and non-parametric tests. The following tests for sample comparison were used: the Kolmogorov–Smirnov test, the t-Student test, the ANOVA test, the Mann–Whitney and the Kruskal–Wallis tests. Statistically significant values were those of *p* < 0.05, while values of *p* < 0.01 were highly significant statistically. Multivariate analysis was carried out by using a binary logistical regression test—Forward LR—and the following variables were used: largest tumor dimension, Clark level, BCC subtype, BCC grade, the cleft’s corresponding tumor nest’s width and the largest tumor nest’s width; significant differences were found between the patients with PNI and/or PCI and those without.

## 3. Results

### 3.1. Patient Group Characteristics

The current study included 244 patients’ cases from the “Sfântul Apostol Andrei” Emergency Clinical Hospital of Galati, Romania. The group was relatively balanced, with almost equal ratios of male and female patients: 51.8% and, respectively, 48.2%. The total of 244 BCC cases included various BCC subtypes, such as superficial, superficial multicentric, nodular, micronodular, infiltrative, morpheaform, basosquamous BCC and Pinkus tumor, as summarized in Table 1. These tumors were located mostly on the head and neck regions (most of them being found on the forehead and the nasal pyramid), but other locations included abdomen (anterior and posterior), thorax (anterior and posterior), shoulder and thigh (Table 2); each subtype’s histopathology characterization is highlighted in Table 3.

### 3.2. Findings

Table 4 highlights the statistical analysis carried out in the current study, with all of the parameters taken into consideration for evaluation and the obtained results. From the patients’ group, approximately half of the BCC patients enrolled in this study did not present with perineural invasion (120), while 49 were positive for PNI, and the rest of them (75) had suggestive signs of PNI (meaning chronic inflammation located perineurally).

**Figure 1:**
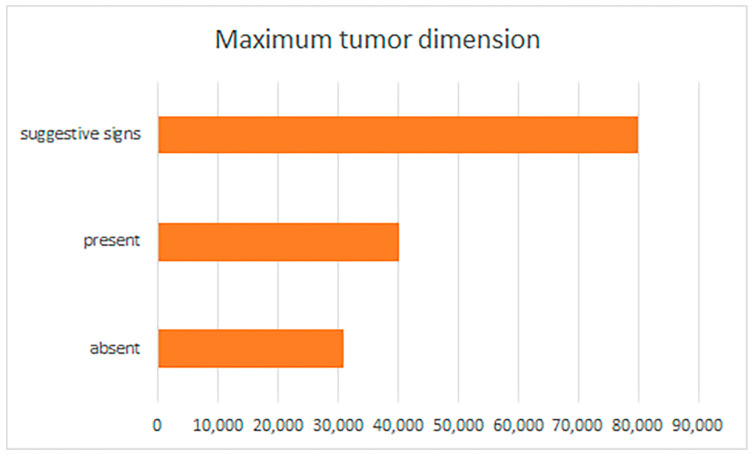
Perineural invasion and suggestive signs, reported by maximum tumor dimension.

BCC patients showing PNI or with suggestive signs of PNI had larger tumors (with median values of 13,683.965 ± 8791.612 µm/13.68 ± 8.79 mm or 11,418.315 ± 10,066.012 µm/11.41 ± 10.06 mm) compared to patients with BCC but without PNI (*p* = 0.000) (Figure 1).

**Figure 2:**
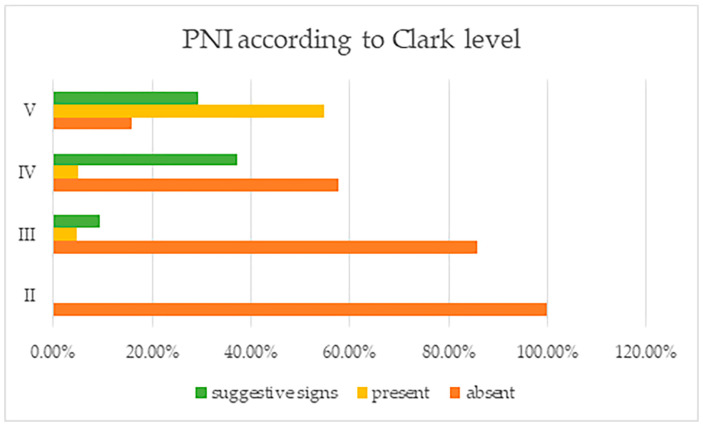
Perineural invasion and suggestive signs, reported by tumor Clark levels.

Concerning the Clark level, tumors with Clark level II were found to not have PNI, a trait which was rather found instead in tumors with other Clark levels (III, IV and V) (Figure 2). In those tumors having Clark level III, the number of patients with PNI was reduced (as opposed to deeper Clark levels), to only 1, but this number increased significantly for tumors with Clark level IV; seven cases were PNI positive, while fifty-one cases had suggestive signs. For Clark level V, more than ½ of cases showed PNI—41—and another 22 cases presented suggestive signs. All of these differences found were, again, statistically significant (*p* = 0.000). Again, there was a parallel increase in PCI with that of the PNI, showing a strong correlation.

**Figure 3:**
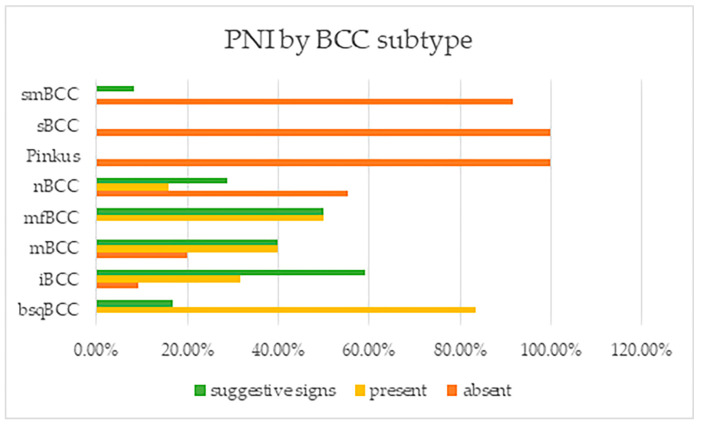
Perineural invasion and suggestive signs reported by BCC subtype. Note: smBCC—superficial multicentric BCC; sBCC—superficial BCC; nBCC—nodular BCC; mfBCC—morpheaform BCC; mBCC—micronodular BCC; iBCC—infiltrative BCC; and bsqBCC—basosquamous BCC.

PNI was found with high frequencies in BCC subtypes that are considered as having an aggressive behavior, being high-risk (Figure 3). PNI was positively identified in all basosquamous BCCs and morpheaform tumors, in a very large amount of infiltrative BCC subtypes and also in micronodular tumors, and in 29 of the nodular ones, being absent, however, in Pinkus/fibroepithelial BCCs or superficial tumors. These differences between tumor subtypes were also found to be statistically significant.

**Figure 4:**
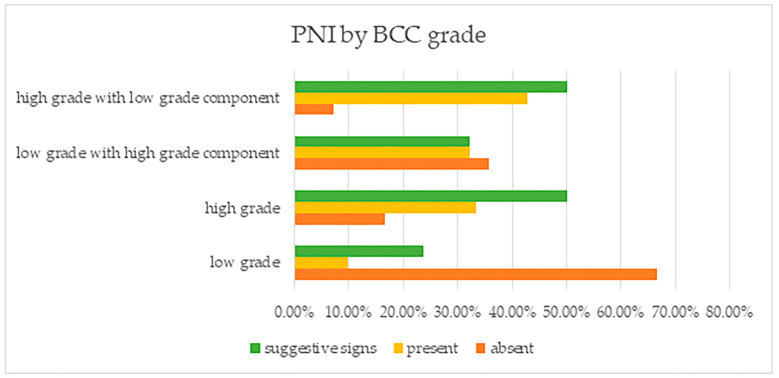
Perineural invasion and suggestive signs, reported by BCC grade.

The finding that PNI was mostly found in high grade tumors (with or without a low grade tumor component) was also statistically significant (*p* = 0.000) (Figure 4). This tumor grade concordance with PNI presence was supported by the fact that patients having low grade tumors did not have PNI (in 96 of cases, PNI was absent), while 41 of those having high grade tumors had PNI (18 patients) or PCI (23 patients).

The multivariate analysis of the PNI prognosis factors needed a three-step constructed model, which had statistical significance (*p* < 0.001), with a sensitivity of 63.3% and a specificity of 92.7%. Of the predictor values used, one in particular had statistical significance; BCC grade, an obvious risk factor. Patients having low-grade BCC, but with a high-grade component, had 3.139 times higher chances of having PNI than those with only low-grade tumors; patients with high grade tumors with a low-grade component had 3.977 times higher chances (than those with low-grade tumors) of PNI. This being said, those with high-grade tumors had 6.512 times the chance of having PNI than those with low-grade ones (Table 5).

The multivariate analysis of the PCI prognosis factors needed a three-step constructed model, which had statistical significance (*p* < 0.001), with a low sensitivity of 5.4% and a high specificity of 97.6%. The model identified two variables as being statistically significant for PCI—the largest tumor dimension and the largest tumor nest (Table 6)—but the associated risks were neutral (OR = 1.000).

## 4. Discussion

PNI is a tumor trait that is (or should be) part of the pathology report when dealing with a BCC. As mentioned earlier, pathologists may not find PNI on the examined slides of a tumor, but they might see suggestive signs for PNI, which consist of PCI (indicating that something is indeed going on with that specific nerve bundle and should be further investigated/reported) [25,26]. What is worrisome for PNI in such cases is that it might be occult, and signs and symptoms can develop even seven and a half years after the initial diagnosis (and after surgical treatment has been implemented) [30].

The current retrospective study took into consideration 244 BCC-suffering patients during the span of 2 years (of 2019 and 2020), with a significantly larger number of patients addressing medical services in 2019, at the beginning of the Coronavirus disease 2019 (COVID-19) pandemic (for which the Severe Acute Respiratory Syndrome Coronavirus-*2* (SARS-CoV-2) was responsible). Patient medical care addressability was significantly lower in 2020 due to the measures taken in order to stop COVID-19 from further spreading with morbidity and mortality-related consequences [31,32,33]. 

This study reported the presence of PNI in 20.1% of cases, and PCI in 30.2% of cases, the remaining numbers not having PNI or PCI. These findings are significantly increased, with the current literature identifying PNI in approximately 1% of cases [34], and some reporting higher incidences of up to 23.1% (closer to our data), a finding dependent on the technique used, conventional pathology or Mohs procedure, and also in connection to larger tumors and to the morpheaform subtype [35,36,37]. Although such ratios have been reported, PNI still remains an underdiagnosed and under-recognized feature of BCCs [38]. The percentages of PNI and PCI parallel one another, and the question of whether such patients should benefit from a more careful follow-up prior to excision is raised. We believe that PCI needs to be considered as a suggestive sign for PNI due to the fact that inflammation in such a location cannot be explained by other processes in the setting of a BCC patient. PCI implies the presence of a local growth; something is developing in this particular place, and the body is reacting to it. The fact that such high numbers of BCC cases recorded the presence of PCI is worrisome and indicative of a much-needed closer patient follow-up, or possibly added treatment options. 

The current study found that PNI and PCI in BCC patients were frequently found in cases of larger tumors with more advanced, deeper Clark levels. Larger tumors tend to have deeper invasion, affecting various skin and soft tissue structures (including nerve fibers) and yielding a deeper Clark level in the pathology report. There was a steady increase in PNI and PCI concerning the BCC cases having large tumor maximum dimension, with the largest tumor recorded as having PCI. With regard to Clark levels, starting from Clark level III, a steady increase in frequency can be noticed, with the highest ratios found in BCC cases having Clark levels IV and V. Larger tumor dimensions and higher Clark levels indicate more aggressive behaviors, a fact also supported by PNI and PCI, inclusively. 

Ratner et al. (2000) and Brown and Perry (2000) reported their findings of PCI and highlighted that tumors with such findings had a more aggressive histologic growth pattern (morfeaform, infiltrative, sclerosing) [20,39], in support of our study. Low grade tumors exhibited PCI and PNI less frequently, as was to be expected from non-aggressive subtypes. The histological subtype is important when dealing with PNI; nodular BCC, although one of the most frequent BCC subtypes, has a low incidence of PNI, while the superficial subtype most frequently has no PNI. 

PNI can be found both in larger nerve bundles and small fibers, but even though there might be histological PNI, there are no clinical symptoms present when small ones are involved. The gross involvement of a nerve might determine the development of pain, paresthesia and hypoesthesia [19,40] or other nerve deficits [41]. A BCC in the head and neck region can affect the trigeminal, facial or even mental nerves [41,42]. Ratner et al. (2000) highlighted that attention must be paid to all nerves affected and that a nerve fiber is not continuously affected by tumor spread, and skip areas exist, with this discontinuous involvement of nerve fibers being explained through an artifactual change during tissue processing by twisting and turning the surgical piece. Finding these discontinuous involvements of nerve fibers and PCI might serve as indicators for a more proximal nerve involvement by the malignant process. As in PNI-positive SCC cases, the presence of PNI requires a more aggressive management (treatment) approach. Whether PNI is present or not has already been regarded as an important prognostic factor, but what about the position of the affected nerve? Lin et al. (2012) have stated that PNI found at the periphery of the tumor might play a prognostic factor role, but insufficient data were evaluated in order to make a concise affirmation. Additionally, the type of PNI might also be important, as a diffuse nerve involvement might indicate a worse prognosis than that of a focal infiltration [43].

Naturally, PNI needs to be differentiated by false-positive tumor aspects. Hassanein et al. (2005) described peritumoral fibrosis with a concentric layering of the fibrous tissue as being a tumor’s stromal trait, which needs to have careful consideration due to the deceiving aspect that it can present with, which mimics PNI. At the same time, they have found this specific peritumoral concentric fibrosis to be a suggestive sign for PNI [44]. By using Mohs micrographic surgery, PNI has been found to be mistaken for other structures such as vessels, erector pili muscles, SCC eddies or granulomatous inflammation [45].

Massey et al. (2020) stated that PNI alone is not a factor of outcome influence, being of relatively low importance, especially in comparison with other parameters that indicate high-risk such as increased tumor diameter, aggressive or high-risk BCC subtype, face location and deep invasion [46], parameters which were reported in our study as being linked to PNI and PCI. This being said, we believe that PNI and PCI are indicators for a more aggressive tumor which can extend beyond the surgical margins. Careful follow-up should be ensured in such patients, and these two parameters should be mentioned in every pathology report, being of help in the patient’s evolution and outcome; a positive PNI diagnosis may help improve treatment interventions, and help with the patient’s morbidity and mortality rates [47]. PNI is indeed an indicator for BCC’s increased morbidity [48]. PNI has been found in larger tumors, and in malignancies which have a higher subclinical extension and higher rates of recurrence, with a significantly increased risk of metastasis. PNI is a tumor trait which was frequently reported as being found in re-excision specimens (Bechert and Stern, 2010) [48,49]. This statement might, in fact, not refer to a real PNI, but a misdiagnosis due to an increased bias towards PNI and due to the nature of the process, which might be a reactive one. The reactive epithelia from adjacent eccrine sweat glands might insinuate into the perineural space and give a false indication of PNI. Although the latter statements disagree with the probability of finding higher incidences of PNI in re-excision specimens, the latter might also be explained by a multifactorial array of other factors: re-excision specimens which might offer an increased amount of tissue for the pathologist to evaluate; the sample might involve deeper skin levels; and pathologists might evaluate with more scrutiny the re-excision specimens for PNI, as opposed to the initial biopsies/excision specimens taken; re-excision specimens might also be a sample taken from a recurrent tumor, rather than a completion of a previous surgical procedure [50]. PNI is a clue for possible future neurological deficits [49], and also for a possible tumor extension with orbital invasion (when dealing with tumors that are site-specific) [51]. A positive PNI diagnosis in a BCC patient is a powerful indicator for long-term patient monitoring, along with obtaining a 5-year local control by combining two treatment options—the surgical one (excision with clear surgical margins) and radiotherapy [52]. PNI in non-melanoma skin cancers such as BCC is a sign for increased rates of local and regional recurrences, and also for diminished disease-free survival periods [53]. Radiotherapy is indicated in BCC cases with PNI (another beneficial aspect of PNI reporting), indicating the possibility of a residual tumor, but the probability of a total cure is reduced as the tumor advances towards the central nervous system [54,55]. An early and accurate PNI diagnosis, with an appropriate risk stratification and a concise and clear patient management strategy, is needed for improving outcomes. 

A retrospective study carried out by Adams et al. in 2020 on two databases from Australia regarding the treatments and outcomes in keratinocyte cancers with PNI has found that, in cases of BCCs with incidental PNI, the surgical approach is a suitable treatment option, on the condition that it has tumor-free margins of at least 3 mm (peripheral tumor margin) and perineural tumor margins of at least 5 mm. BCC lesions having PNI that involved nerve bundles of under 0.1 mm in diameter were more frequently treated using the surgical approach, while those tumors involving nerves of at least 0.1 mm in diameter were also associated with adjuvant radiotherapy treatment. These treatment approaches allowed physicians to obtain disease-free intervals of 5 years; after 5 years of the initial treatment, no recurrences were found in the BCC patients enrolled in the study [56].

A possible important future research pathway could stem from the possible inhibition of BCC development and/or progression by using non-steroidal anti-inflammatory drugs (NSAIDs), namely cyclooxygenase-2 (COX-2) inhibitors, which the current literature data report as having chemoprotective properties in patients with such pathology [57]. Muranushi et al. (2016) are in support of this statement, with their study reporting a significant risk reduction in BCC development in patients using such drugs, aiding in those high-risk populations that might be at risk for developing BCC [58]. However, conflicting data emerge, as Yen et al. highlighted, in their 2022 study, that the use of COX-2 inhibitors did not diminish the risk of developing skin cancer, and, even more so, it increased the BCC risk [59]. The exact influence that COX-2 inhibitors exert on the BCC’s development and/or progression is not yet fully understood [60,61], but careful consideration needs to be carried out, as such drugs carry the risk of varied, multiple adverse skin reactions (from fixed drug eruptions, exanthema and urticarial, to Stevens-Johnson syndrome, toxic epidermal necrolysis or drug-induced vasculitis) and even systemic ones (hypertension, edema or congestive heart failure) [62,63].

## 5. Conclusions

PCI is considered to be a positive clue for perineural involvement by a malignant tumor, in particular that a nerve fiber/bundle is being affected by a process; something is going on with it, otherwise inflammation would not be present. PCI and PNI were frequently found in specific patient and tumor settings, such as in aggressive BCC subtypes and large, high-grade tumors with deeper Clark levels, reflecting the literature and adding updated data. PCI and PNI are important clues for patient prognosis, and they should both be reported by pathologists, giving treating clinicians a more exact view on the tumor’s behavior and influencing treatment options to further ensure a positive patient outcome.

## Figures and Tables

**Table 1:** Gender distribution of the BCC subtype.

Subtype of BCC	Sex				Total		Pearson
	M		F				Chi-Squared
	N	%	N	%	N	%	
Infiltrative	15	12.1	7	5.8	22	9.0	Chi^2^ = 14,252
Basosquamous	5	4.0	1	0.8	6	2.5	with a *p* value of 0.047
Micronodular	3	2.4	1	0.8	4	1.6	
Morpheaform	9	7.3	6	5.0	15	6.1	
Nodular	88	71.0	93	77.5	181	74.2	
Pinkus tumor	1	0.8	0	0	1	0.4	
Superficial multicentric	3	2.4	9	7.5	12	4.9	
Superficial	0	0	3	2.5	3	1.2	
Total	124	100.0	120	100.0	244	100.0	

**Table 2:** BCC tumor location by gender distribution.

Tumor Location	Gender				Total		Pearson
	F		M				Chi-Squared
	N	%	N	%	N	%	
Anterior abdomen			2	1.6%	2	0.8%	Chi^2^ = 12,129
Anterior thorax	4	3.3%	2	1.6%	6	2.5%	with a *p* value of 0.841
Arm			1	0.8%	1	0.4%	
Auricle	8	6.7%	10	8.1%	18	7.4%	
Calf	1	0.8%	1	0.8%	2	0.8%	
Cheek	15	12.5%	16	12.9%	31	12.7%	
Chin	4	3.3%	1	0.8%	5	2.0%	
Eyelid	12	10.0%	12	9.7%	24	9.8%	
Forearm			1	0.8%	1	0.4%	
Forehead	22	18.3%	23	18.5%	45	18.4%	
Lip comissure	8	6.7%	5	4.0%	13	5.3%	
Nasal pyramid	23	19.2%	22	17.7%	45	18.4%	
Neck	5	4.2%	6	4.8%	11	4.5%	
Posterior abdomen	2	1.7%	1	0.8%	3	1.2%	
Posterior thorax	9	7.5%	8	6.5%	17	7.0%	
Scalp	5	4.2%	11	8.9%	16	6.6%	
Shoulder	1	0.8%	1	0.8%	2	0.8%	
Thigh	1	0.8%			1	0.4%	
Upper lip			1	0.8%	1	0.4%	
Total	120	100.0%	124	100.0%	244	100.0%	

**Table 3:** Summary of BCC subtype histology traits.

Subtype of BCC	Histopathology
Pinkus tumor	Anastomosing strands of basaloid cells in a fibrous stroma
Superficial	Multiple, small islands of basaloid cells that descend from the epidermis without dermal invasion
Nodular	Centrally haphazard arrangement with peripheral palisading cells forming tumor islands ± ulceration
Micronodular	~ Nodular subtype, but small nodules, increased risk for recurrence
Morpheaform	Thin strands of basaloid cells with dermal invasion, in a dense fibrous/collagenous stroma
Infiltrative	Thin strands of basaloid cells invading the dermis, without collagenous stroma (sometimes overlapping with morpheaform)
Basosquamous	Nests/strands of cells which mature to larger, paler cells, with noperipheral palisading (features common to BCC and SCC)

Refer to [1,29].

**Table 4:** Study parameters’ evaluation and statistical analysis results.

**Pn1**	**Type of BCC—Pearson Chi^2^ = 55.940 with a *p* Value of 0.000**								
	bsqBCC	iBCC	mBCC	mfBCC	nBCC	Pinkus tumor	sBCC	smBCC	Total
Present	5	7	6	2	29	0	0	0	49
Suggestive signs	1	13	6	2	52	0	0	1	75
Absent	0	2	3	0	100	1	3	11	120
	**Clark Level—Pearson Chi^2^ = 103.278 with a *p* Value of 0.000**								
	II	III	IV	V	Total				
Present		1	7	41	49				
Suggestive signs		2	51	22	75				
Absent	11	18	79	12	120				
	**BCC Grade—Pearson Chi^2^ = 55.313 with a *p* Value of 0.000**								
	Low grade	High grade	Low grade with high grade component	High grade with low grade component	Total				
Present	14	6	17	12	49				
Suggestive signs	34	9	17	14	75				
Absent	96	3	19	2	120				
	**Largest Tumor Dimension in Micrometers—** **Kruskal–Wallis, with a *p* Value of 0.000**								
	Cases	Standard deviation	Mean	Standard error of mean	Median	Maximum		Minimum	
Present	49	8791.612	13,683.965	1255.944	12,034.50	40,200.0		1055.70	
Suggestive signs	75	10,066.012	11,418.315	1162.323	8765.50	80,000.0		1108.70	
Absent	120	5724.653	8463.822	522.587	7057.25	31,022.0		755.50	

Abbreviations: bsqBCC, basosquamous basal cell carcinoma; iBCC, infiltrative BCC; mBCC, micronodular BCC; mfBCC, morfeaform BCC; nBCC, nodular BCC; sBCC, superficial BCC; smBCC, superficial multicentric BCC.

**Table 5:** PNI multivariate analysis by BCC grade.

	B	*p*-Value	OR	95% C.I. for OR	
				Lower	Upper
Low-grade BCC		0.016			
Low-grade BCC with high-grade component	1.144	0.027	3.139	1.137	8.669
High-grade BCC with low-grade component	1.381	0.020	3.977	1.246	12.692
High-grade BCC	1.874	0.014	6.512	1.452	29.208

**Table 6:** PCI multivariate analysis.

	B	*p*-Value	OR	95% C.I. for OR	
				Lower	Upper
Largest tumor dimension	0.000	0.044	1.000	1.000	1.000
Largest tumor nest	0.000	0.007	1.000	0.999	1.000

## Data Availability

The data presented in this study are available on request from the corresponding author.
